# Supplementary figures and images for: Full-length direct RNA sequencing uncovers stress granule-dependent RNA decay upon cellular stress
Source: eLife. 2024 Dec 19;13:RP96284. doi: 10.7554/eLife.96284 (PMC11658763; doi:10.7554/eLife.96284)

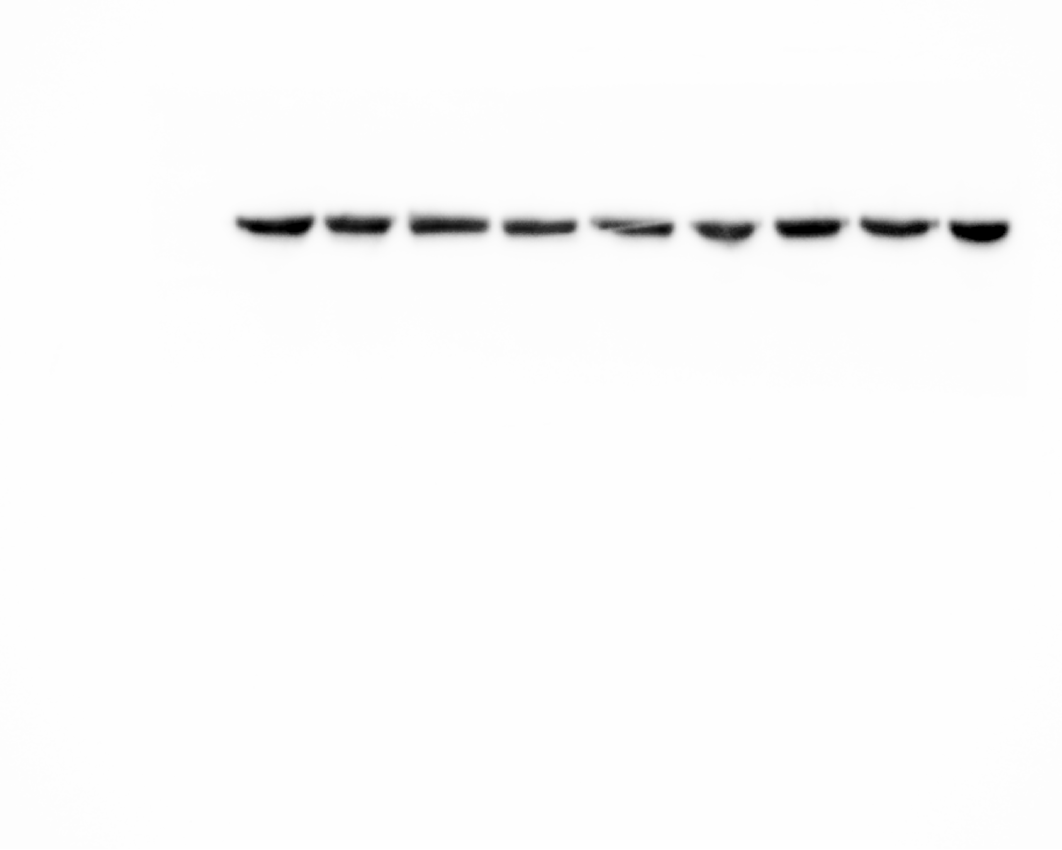

Supplement: Figure 3—source data 1. [file elife-96284-fig3-data1.zip › ACTB/sm 2021-11-02 11h35m16s(Chemiluminescence).tif]

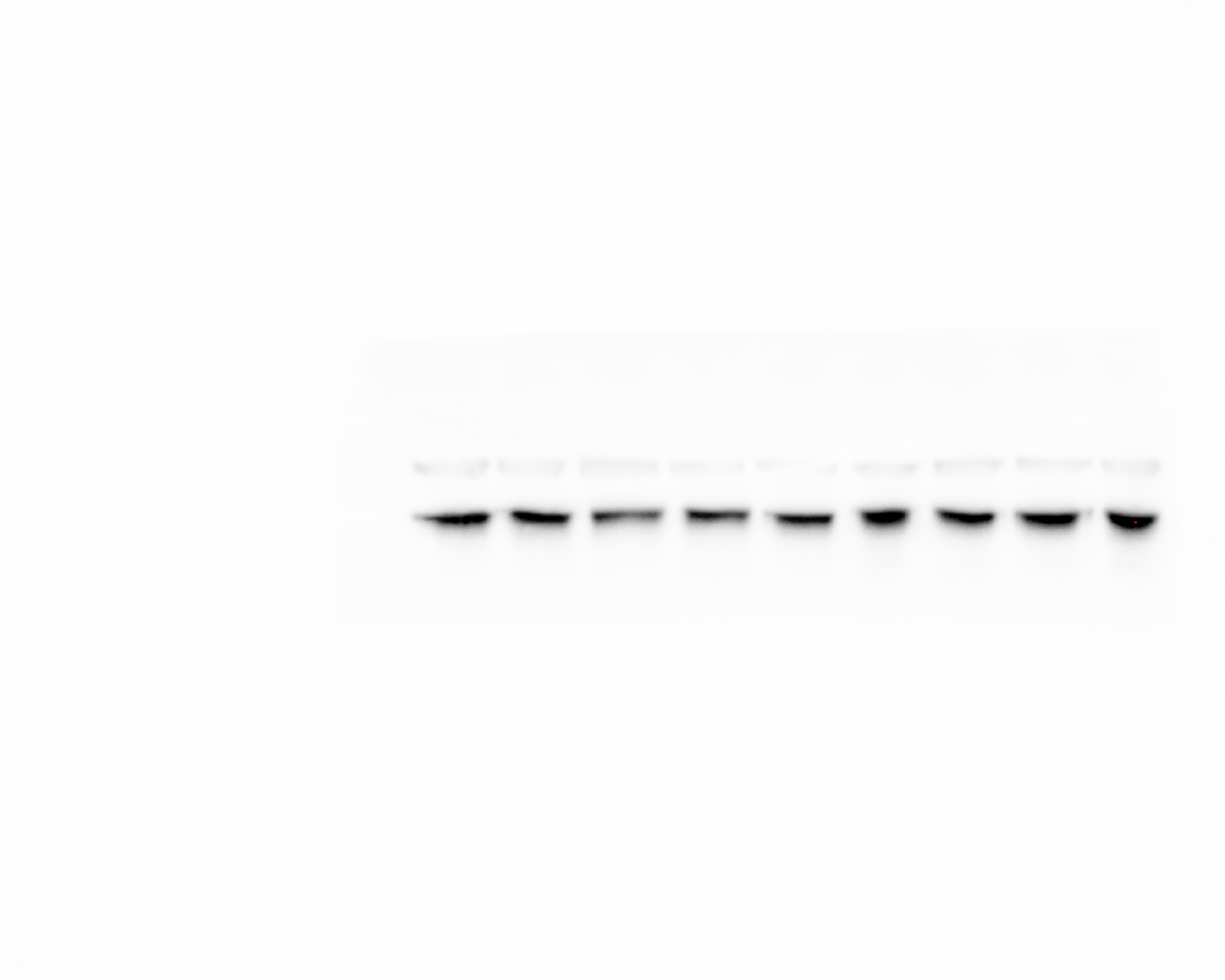

Supplement: Figure 3—source data 1. [file elife-96284-fig3-data1.zip › eif2a/sm 2021-11-03 11h05m30s(Chemiluminescence).tif]

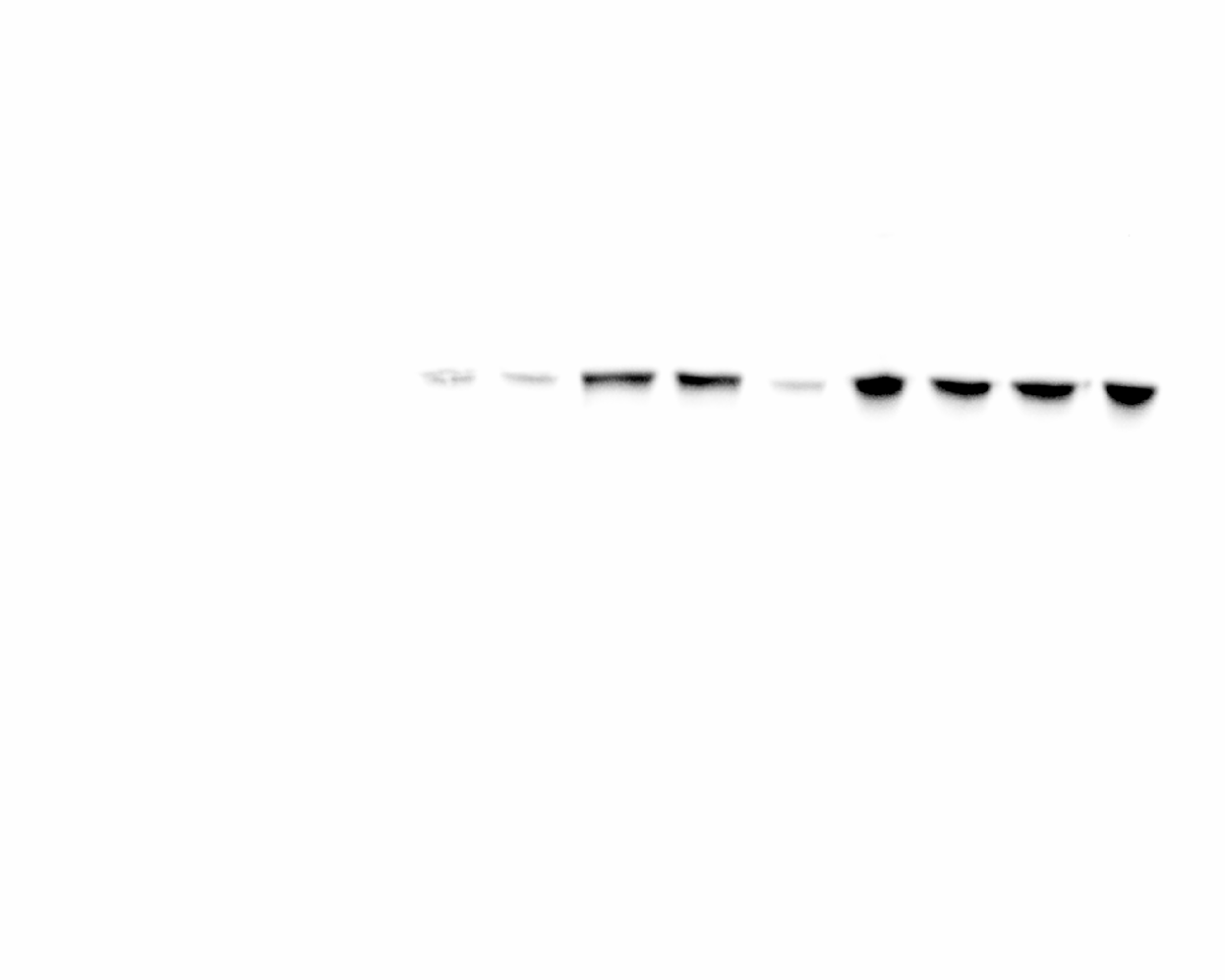

Supplement: Figure 3—source data 1. [file elife-96284-fig3-data1.zip › eif2a-p/sm 2021-11-01 18h02m25s(Chemiluminescence).tif]

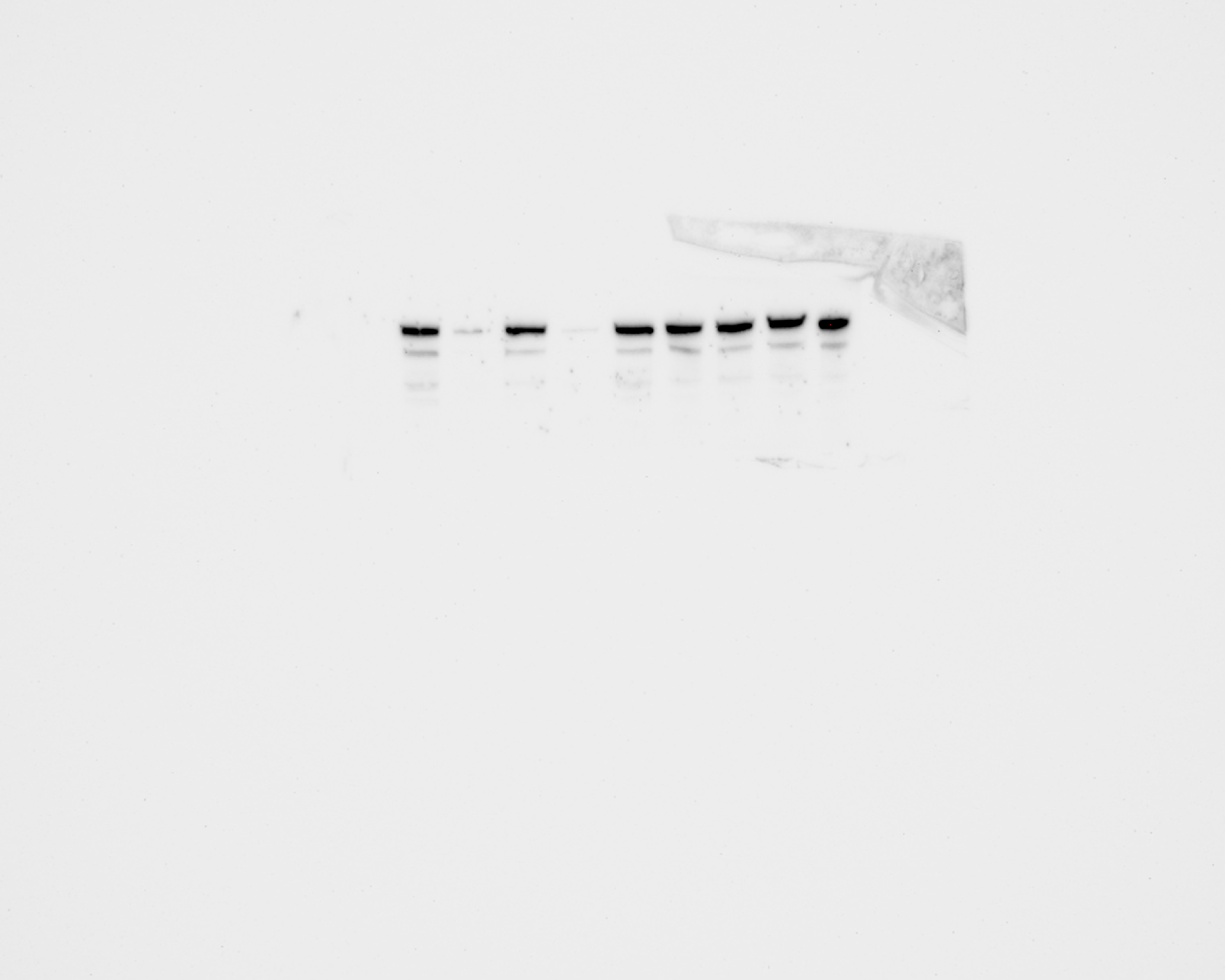

Supplement: Figure 3—source data 1. [file elife-96284-fig3-data1.zip › XRN1/sm 2021-11-01 17h41m15s(Chemiluminescence).tif]

**Figure 3a**

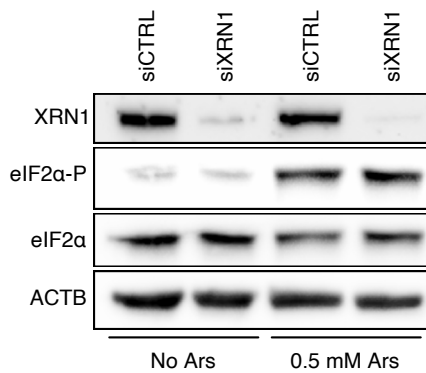

**Raw blots:**

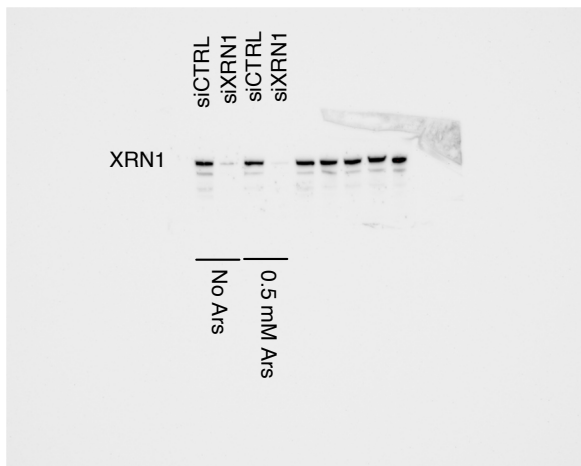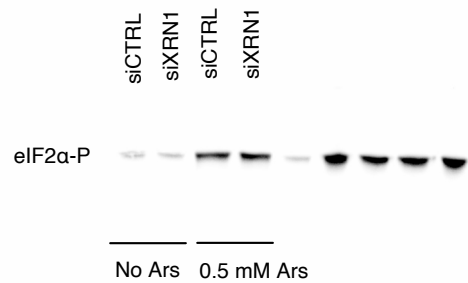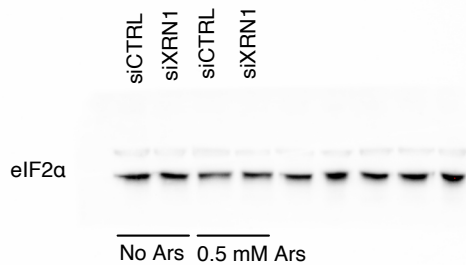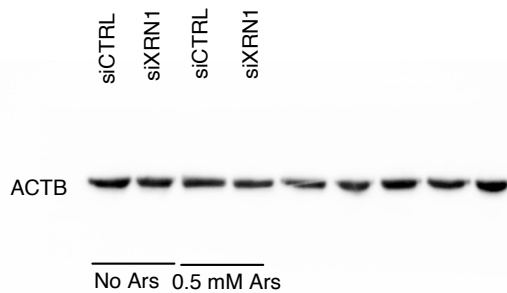

Supplement: Figure 3—source data 2. [file elife-96284-fig3-data2.zip › Figure 2-source data 2.pdf]

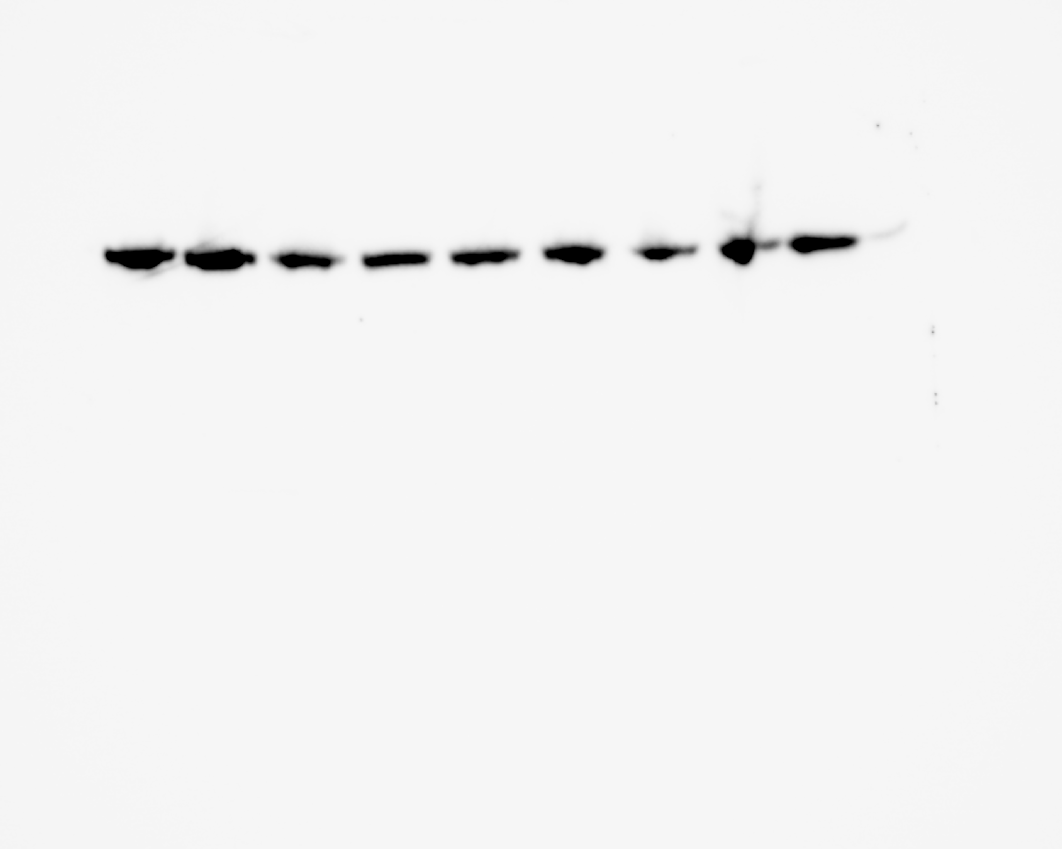

Supplement: Figure 3—figure supplement 1—source data 1. [file elife-96284-fig3-figsupp1-data1.zip › ACTB/cedric 2021-07-06 13h45m06s 4.939s 0.004s(Chemiluminescence).tif]

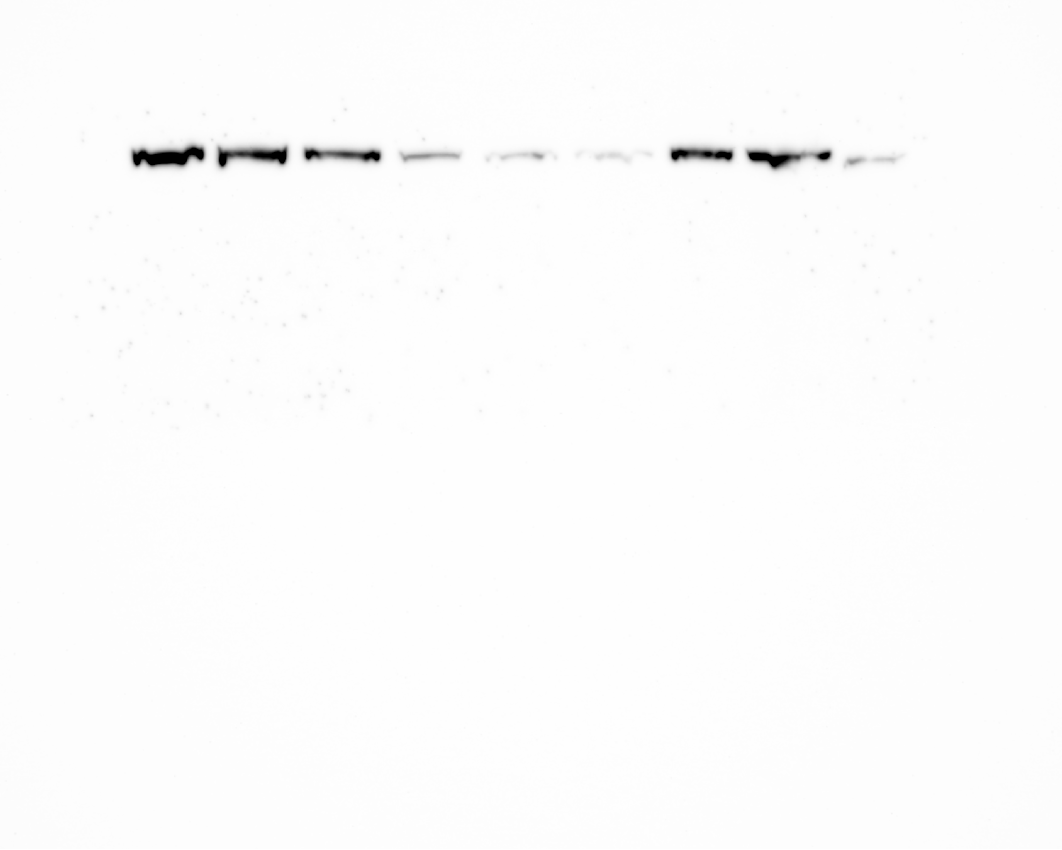

Supplement: Figure 3—figure supplement 1—source data 1. [file elife-96284-fig3-figsupp1-data1.zip › XRN1/cedric 2021-07-06 14h15m37s 300.000s 0.003s(Chemiluminescence).tif]

Supplementary Figure 3a

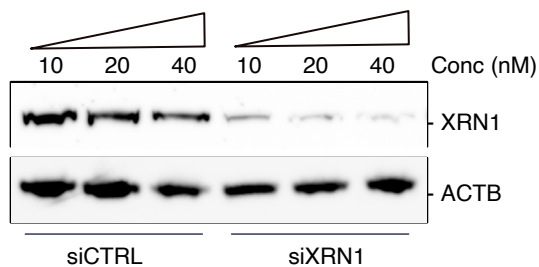

**Raw blots:**

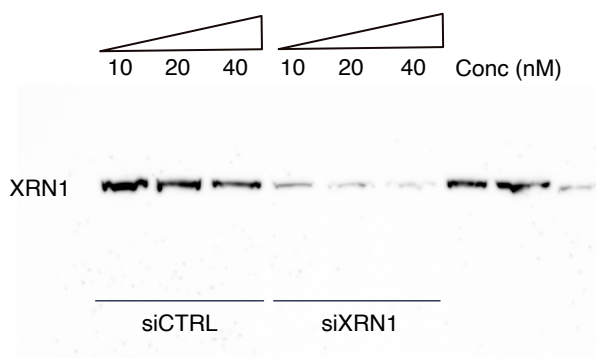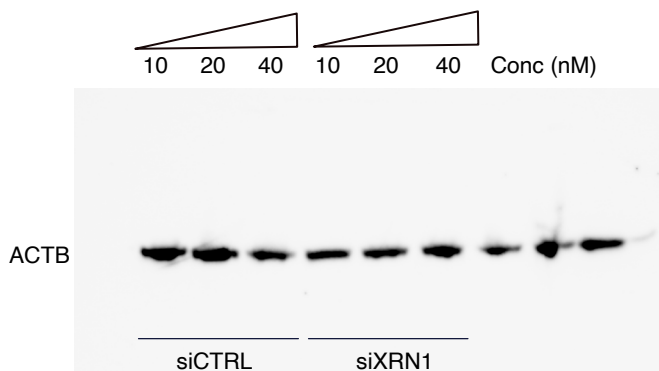

Supplement: Figure 3—figure supplement 1—source data 2. [file elife-96284-fig3-figsupp1-data2.zip › Figure 5-figure supplement 1-source data 2.pdf]

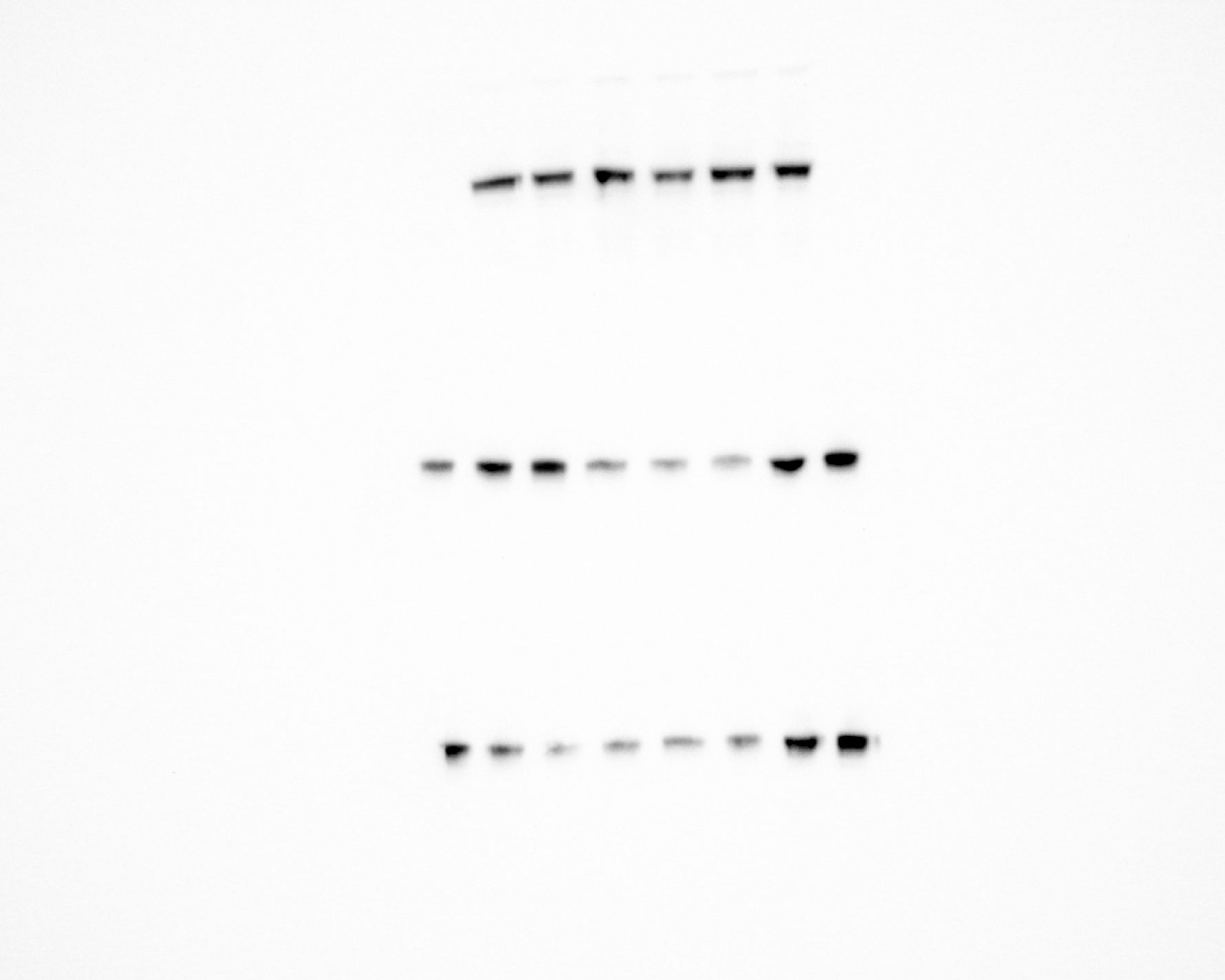

Supplement: Figure 5—source data 1. [file elife-96284-fig5-data1.zip › panel a/eif2a/sm 2021-10-13 08h37m47s(Chemiluminescence).tif]

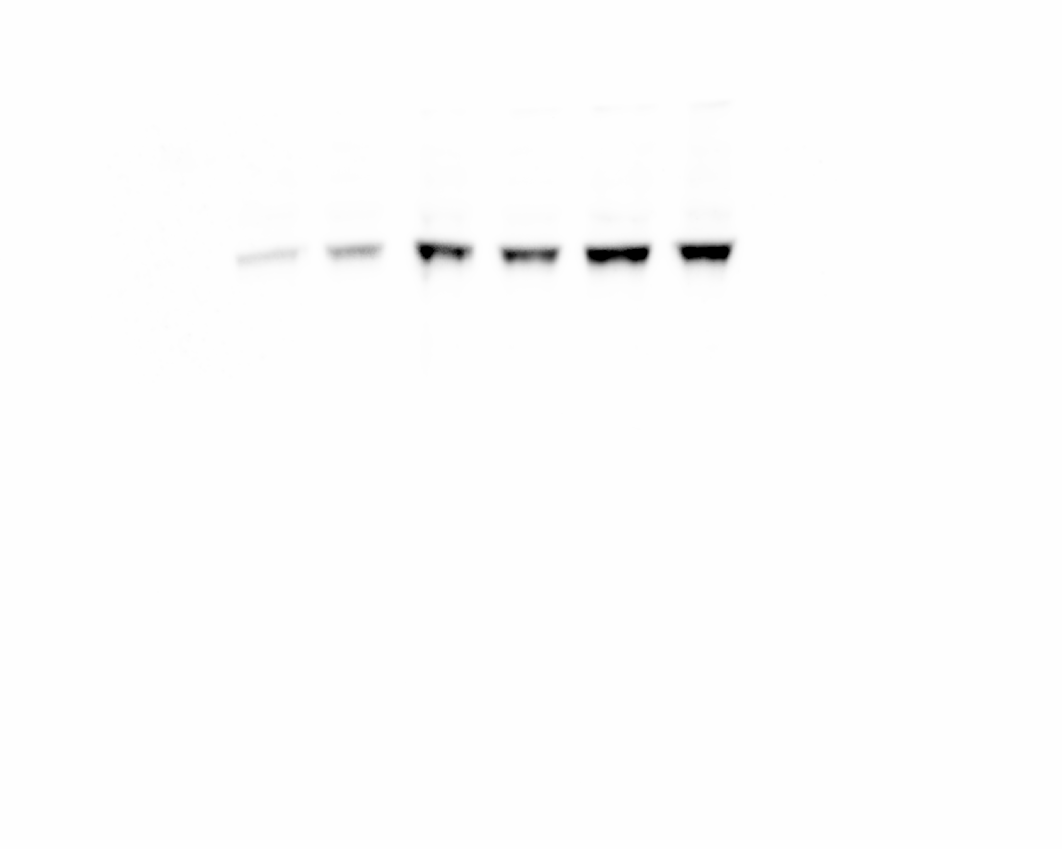

Supplement: Figure 5—source data 1. [file elife-96284-fig5-data1.zip › panel a/eif2a-p/sm 2021-10-08 14h10m28s(Chemiluminescence).tif]

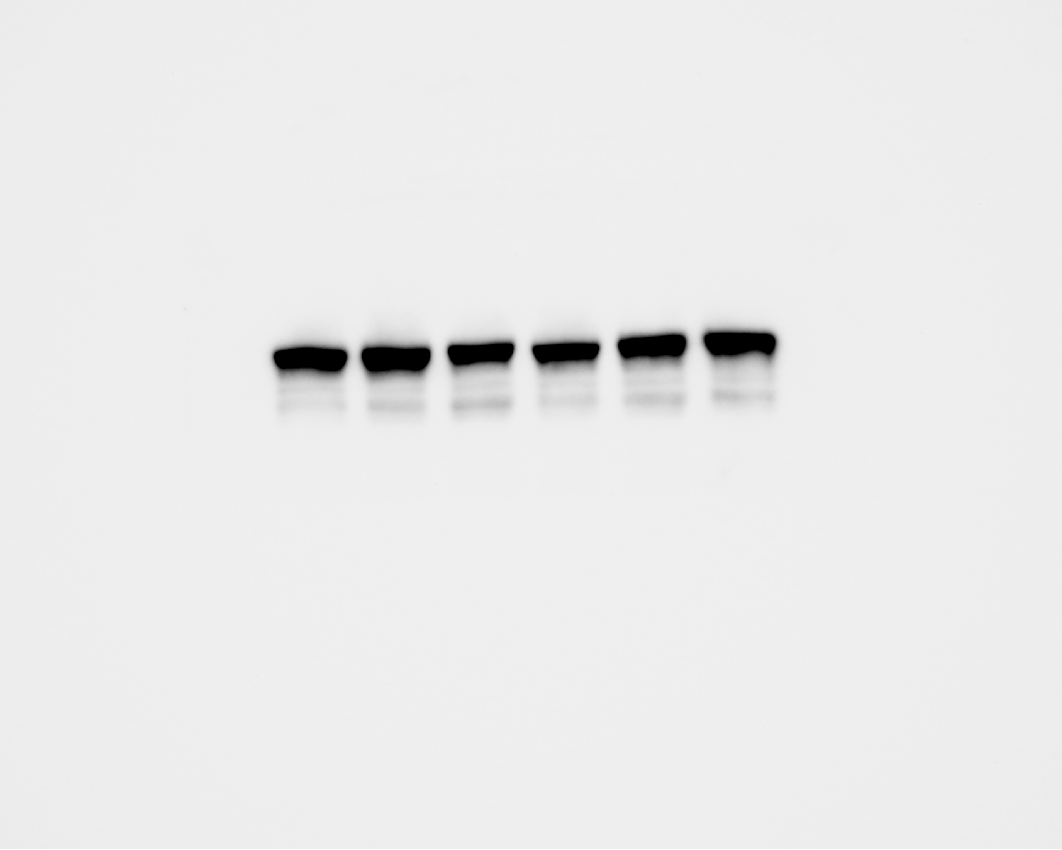

Supplement: Figure 5—source data 1. [file elife-96284-fig5-data1.zip › panel b/eif2a/sm 2021-10-26 14h29m22s(Chemiluminescence).tif]

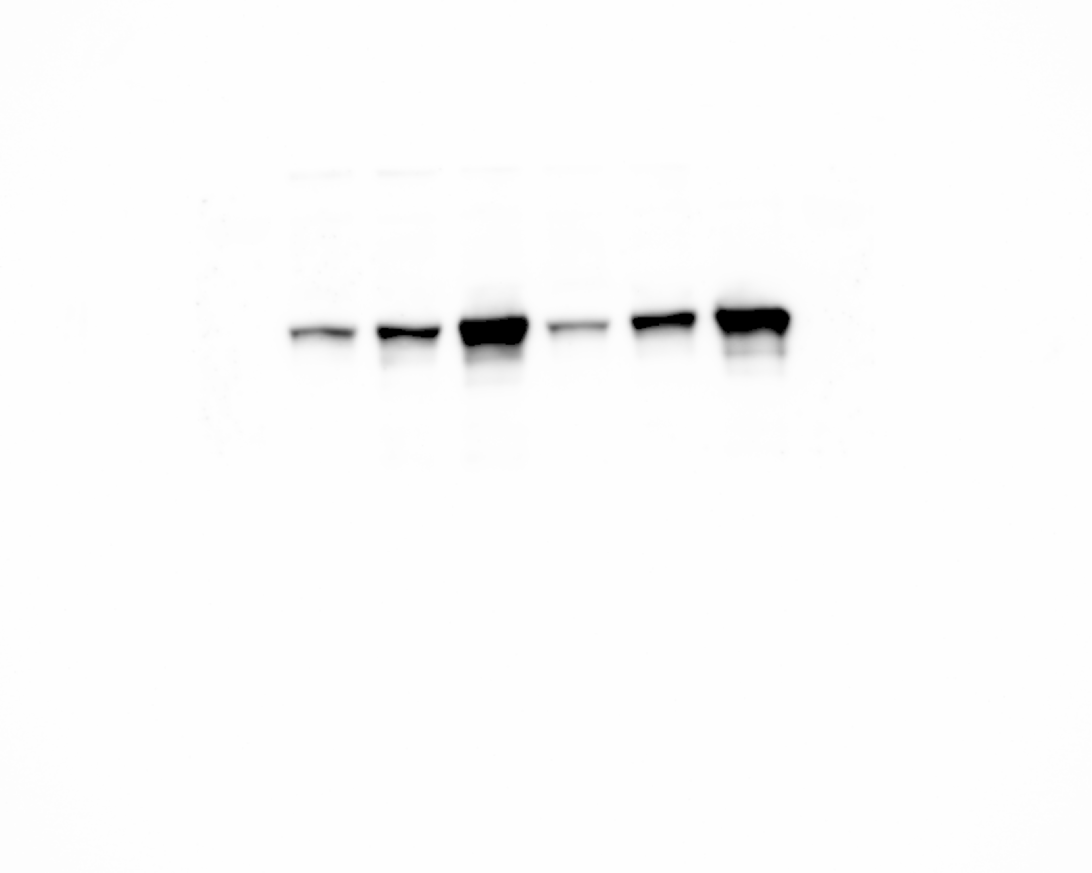

Supplement: Figure 5—source data 1. [file elife-96284-fig5-data1.zip › panel b/eif2a-p/sm 2021-10-22 12h17m28s(Chemiluminescence).tif]

**Figure 5****a**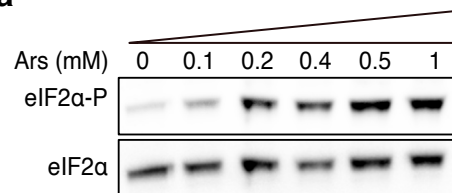**b**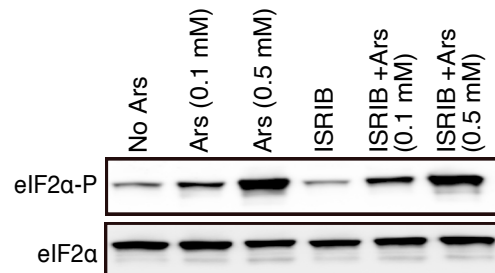**Raw blots:**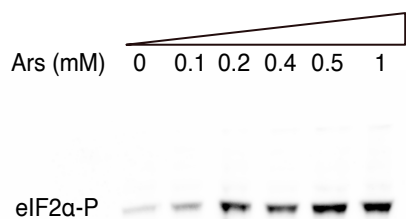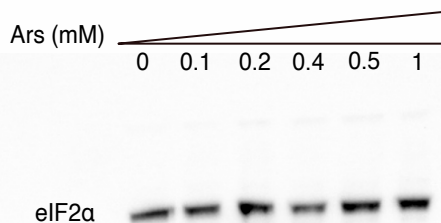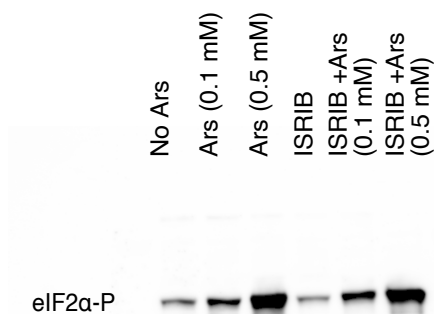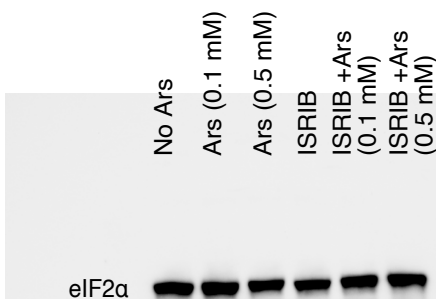

Supplement: Figure 5—source data 2. [file elife-96284-fig5-data2.zip › Figure 5-source data 2.pdf]
